# Molecularly imprinted polymers as selective adsorbents for ambient plasma mass spectrometry

**DOI:** 10.1007/s00216-017-0281-2

**Published:** 2017-03-20

**Authors:** Michał Cegłowski, Marek Smoluch, Edward Reszke, Jerzy Silberring, Grzegorz Schroeder

**Affiliations:** 1 Faculty of Chemistry, Adam Mickiewicz University in Poznan, Umultowska 89b, 61-614 Poznań, Poland; 2 Department of Biochemistry and Neurobiology, Faculty of Materials Science and Ceramics, AGH University of Science and Technology, Al. Mickiewicza 30, 30-059 Kraków, Poland; 3 ERTEC-Poland, Rogowska 146/5, 54-440 Wrocław, Poland

**Keywords:** Molecularly imprinted polymers, Ambient plasma mass spectrometry, Selective adsorption, Molecular scavengers, Flowing atmospheric pressure afterglow

## Abstract

The application of molecularly imprinted polymers (MIPs) as molecular scavengers for ambient plasma ionization mass spectrometry has been reported for the first time. MIPs were synthesized using methacrylic acid as functional monomer; nicotine, propyphenazone, or methylparaben as templates; ethylene glycol dimethacrylate as a cross-linker; and 2,2′-azobisisobutyronitrile as polymerization initiator. To perform ambient plasma ionization experiments, a setup consisting of the heated crucible, a flowing atmospheric-pressure afterglow (FAPA) plasma ion source, and a quadrupole ion trap mass spectrometer has been used. The heated crucible with programmable temperature allows for desorption of the analytes from MIPs structure which results in their direct introduction into the ion stream. Limits of detection, linearity of the proposed analytical procedure, and selectivities have been determined for three analytes: nicotine, propyphenazone, and methylparaben. The analytes used were chosen from various classes of organic compounds to show the feasibility of the analytical procedure. The limits of detections (LODs) were 10 nM, 10, and 0.5 μM for nicotine, propyphenazone, and methylparaben, respectively. In comparison with the measurements performed for the non-imprinted polymers, the values of LODs were improved for at least one order of magnitude due to preconcentration of the sample and reduction of background noise, contributing to signal suppression. The described procedure has shown linearity in a broad range of concentrations. The overall time of single analysis is short and requires ca. 5 min. The developed technique was applied for the determination of nicotine, propyphenazone, and methylparaben in spiked real-life samples, with recovery of 94.6–98.4%. The proposed method is rapid, sensitive, and accurate which provides a new option for the detection of small organic compounds in various samples.

**Electronic supplementary material:**

The online version of this article (doi:10.1007/s00216-017-0281-2) contains supplementary material, which is available to authorized users.

**Graphical abstract:**
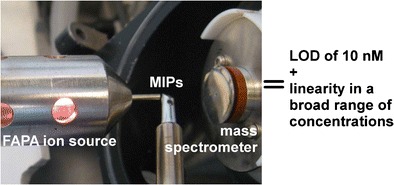
The experimental setup used for analysis

## Introduction

Functional polymers form a wide group of materials that possess unique physicochemical properties. They find numerous applications in various areas, such as drug delivery [1], optoelectronics [2], catalysis [3], water purification [4, 5], preparation of membranes [6], and stimuli-responsive polymers [7]. Molecularly imprinted polymers (MIPs) form a unique group of functional polymers due to their antibody-like affinity. They are obtained during copolymerization of the functional monomers and cross-linkers in the presence of template molecules. After removal of the templates, MIPs possess recognition cavities that are complementary to the template molecules in terms of shape, size, and location of functional groups. In contrary to the natural antibodies, MIPs show many outstanding advantages, such as high chemical stability, excellent reusability, relatively easy, reproducible, and low-cost synthesis [8]. As a result, MIPs have been widely used as artificial receptors for separation purposes, as sensors, to promote catalysis, during drug development and for screening [9–11]. One of the most widely used application of MIPs is solid-phase extraction (SPE). SPE is one of the most popular sample pretreatment methods nowadays, because it allows for the concentration and isolation of an analyte from various complex matrices [12]. The high affinity and selectivity of MIPs is ideally suited for their applications in SPE [13–20].

Ambient ionization mass spectrometry (ambient MS) comprises a group of various techniques, which allows for MS analysis of various samples at atmospheric pressure. They provide rapid, direct, and high-throughput analyses with no or only minimal sample pretreatment. Moreover, substances can be analyzed directly from surfaces or other matrices [21]. Plasma-based techniques, particularly direct analysis in real-time (DART) [22], flowing atmospheric-pressure afterglow (FAPA) [23–25], low-temperature plasma (LTP) [26], dielectric barrier discharge ionization (DBDI) [27–29], and various types of microplasma [30], form a unique group among ambient MS techniques. They involve the generation of a direct current or radiofrequency electrical discharge between a pair of electrodes in contact with a flowing inert gas, creating a stream of ionized molecules, radicals, excited state neutrals, and electrons. The obtained plasma species are directed towards the sample, thus resulting in desorption and ionization of the analytes. Ambient plasma MS techniques have the advantages due to their simple instrumentation, rugged construction, absence of solvents, and generation of singly charged analyte species that are easily identifiable than multiple-charged ions and various adducts produced by the spray-based techniques [31].

The combination of spray-based ambient MS and MIPs films glued to the stainless steel probe has been reported by Figueiredo et al. [32]. The researchers described the use of an easy, ambient sonic-spray ionization mass spectrometry (EASI-MS) for the analysis of phenothiazines extracted from the urine by MIPs. Van Biesen et al. have reported the use of desorption electrospray ionization-mass spectrometry (DESI-MS) coupled with thin-film MIPs for selective extraction and quantification of 2,4-dichlorophenoxyacetic acid [33].

In this study, MIPs selective towards nicotine, propyphenazone, and methylparaben (template molecules used for MIPs synthesis) were obtained. The analytes used were chosen from various classes of organic compounds to show the feasibility of the analytical procedure. The physicochemical properties and adsorption characteristics of the obtained MIPs were examined. After adsorption of particular analytes, MIPs were transferred to the programmably heated crucible, which allowed for thermal desorption of these compounds. The vapors obtained during thermal desorption were directly ionized and analyzed using FAPA mass spectrometry. In contrary to other published designs, we have not used MIPs films but conventional, bulk MIPs. To the best of our knowledge, this is the first study describing a combination of MIPs and plasma-based ambient mass spectrometry. The advantage of using bulk MIPs over MIPs films is the possibility to introduce more analyte to the plasma source which can improve sensitivity of the analytical method.

## Materials and methods

### Chemicals and reagents

All reagents used were commercial products. Methacrylic acid, ethylene glycol dimethacrylate (EGDMA), 2,2′-azobisisobutyronitrile solution (0.2 M in toluene), nicotine, propyphenazone, and methylparaben were obtained from Sigma-Aldrich (St. Louis, MO, USA). All solvents were of the p.a. grade, obtained from Avantor Performance Materials Poland S.A. (Gliwice, Poland) and were used without further purification.

### Urine and plasma samples

Drug-free urine samples used for the experiments involving real-life samples were obtained from the laboratory staff volunteers. Human plasma samples were obtained from Sigma-Aldrich (St. Louis, MO, USA).

### Instrumentation

The surface morphology of the polymer particles was examined using the scanning electron microscope (SEM, Hitachi S3000N, Hitachi Co., Ltd., Tokyo, Japan). The FT-IR (Fourier transform infrared) spectra were obtained using the Nicolet iS 50 FT-IR spectrometer (Thermo Scientific, Waltham, MA, USA). UV–vis measurements were made with the aid of an Agilent 8453 (Santa Clara, California, US) spectrophotometer using 1-cm plastic cuvettes. Mass spectra were obtained using the NOVA011 (ERTEC, Wroclaw, Poland) FAPA ambient plasma source combined with a Bruker Esquire 3000 ion trap mass spectrometer (Bruker Daltonics, Bremen, Germany). The experimental details of the plasma ion source were described in our previous publications [34–36].

### Synthesis of MIPs and NIPs

**Fig. 1:**
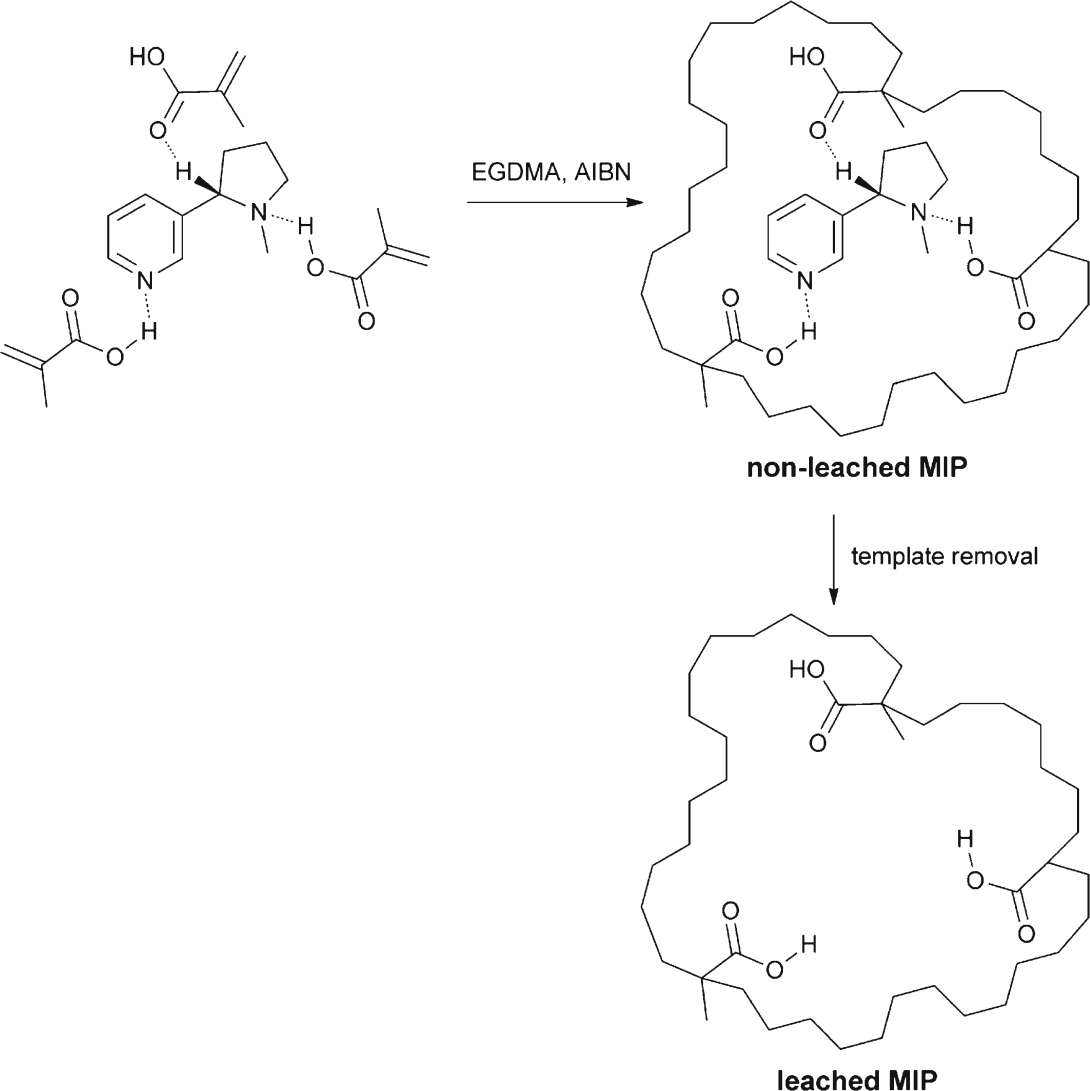
Scheme of MIP(nic) synthesis

All molecularly imprinted polymers were synthesized using the procedure which has been proven to result in a synthesis of water-compatible, molecularly imprinted polymers [37, 38]. Firstly, 1 mmol of the template (nicotine, propyphenazone, or methylparaben) and 4 mmol of methacrylic acid were dissolved in 20 mL chloroform in a glass pressure tube. The solution was sonicated and purged with nitrogen for 30 min. Afterwards, 20 mmol EGDMA and 2 mL 2,2′-azobisisobutyronitrile solution (0.2 M in toluene) were added. The tube was sonicated and purged with nitrogen for 10 min, sealed, and placed in an oven for 18 h at 60 °C. After polymerization, the polymer was dried under reduced pressure, grounded using mortar and pestle, and sieved using a 60-mesh sieve. The removal of template molecules from MIPs was accomplished through a Soxhlet extraction. A sample of MIPs was placed inside the cellulose extraction thimble. The extraction solvent was a mixture of ethanol and acetic acid (9:1 *v*/*v*). The extraction was continued for 24 h, whereas the heating power was adjusted to allow the solvent to cycle every 30 min. Afterwards, the Soxhlet extraction was repeated using pure ethanol as an extraction solvent. Finally, the materials obtained were dried under reduced pressure. As a result, three MIPs were obtained: imprinted with nicotine (denoted as MIP(nic)), propyphenazone (MIP(prph)), and methylparaben (MIP(mpb)). The exemplary scheme of synthesis using nicotine as a template molecule is presented in Fig. 1. The schemes of synthesis using propyphenazone and methylparaben as template molecules have been presented in Electronic Supplementary Material (ESM) Figs. S1 and S2, respectively. A non-imprinted polymer (NIP) was synthesized similarly to the MIPs but without the use of template molecules.

### Adsorption experiments

The adsorption of nicotine, propyphenazone, or methylparaben on the corresponding MIPs and NIP was examined using batch experiments. To prepare adsorption isotherms, a series of samples containing 5 mg of an appropriate MIPs or NIP was equilibrated with 2 mL solution containing various concentrations (0.01–1 mM) of nicotine, propyphenazone, or methylparaben. The solution was shaked at room temperature for 24 h. After adsorption, the mixture was isolated by centrifugation and examined with UV–vis spectrophotometer at 261 nm for nicotine, 266 nm for propyphenazone, and 256 nm for methylparaben. The precise concentration of all analytes was measured by plotting calibration curves. The adsorption amount (*q*_e_; mg g^−1^) was calculated from the following equation:

1 $$ {q}_{\mathrm{e}}=\frac{\left({C}_0-{C}_{\mathrm{e}\mathrm{q}}\right) V}{m} $$

where *C*_0_ and *C*_eq_ are the initial and equilibrium concentrations (mg L^−1^), *m* is the sorbent mass (g), and *V* is the solution volume (L).

For the adsorption kinetic studies, 5 mg of an appropriate MIPs or NIP and 2 mL of solution containing nicotine, propyphenazone, or methylparaben at the initial concentration of 0.1; 0.01, and 0.05 mM, respectively, were stirred at room temperature. The concentration of analytes was measured at preset time intervals using UV–vis spectrophotometer. The amount of adsorbed material at time t, q_t_ (mg g^−1^) was calculated from:

2 $$ {q}_{\mathrm{t}}=\frac{\left({C}_0-{C}_{\mathrm{t}}\right) V}{m} $$

where *m* is the sorbent mass (g), *C*_0_ is the initial concentration, *C*_t_ is the concentration at time *t* (h), and *V* is the solution volume (L).

To prepare MIPs and NIP for FAPA-MS experiments, 10 mg of MIPs or NIP were shaken at room temperature in 10 mL water solution of an appropriate analyte in a broad range of concentrations (from 5 nM to 1 mM) for 12 h. Afterwards, MIPs and NIP were isolated by centrifugation and decantation of the supernatant. One milligram of each polymer was subsequently transferred to the FAPA-MS setup.

Experiments involving the real life samples were performed by spiking urine or plasma with appropriate analytes (0.05–5 μM). The urine was spiked with nicotine [39] or methylparaben [40], as these compounds are excreted from the human body in a free form. The plasma was spiked with propyphenazone. This substance can be found in a free form in the plasma only and is excreted from the human body as various biotransformation products [41]. To adsorb analytes from these samples 5 mg of the appropriate MIPs or NIP were shaken at room temperature in 5 mL of spiked urine or plasma sample. Afterwards, MIPs and NIP were isolated by centrifugation and decantation of the supernatant. One milligram of each polymer was subsequently transferred to the FAPA-MS setup.

### FAPA-MS experiments

**Fig. 2:**
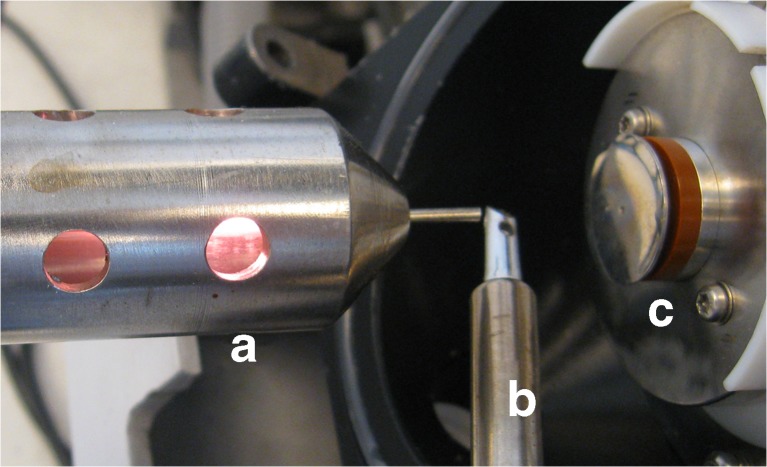
The setup used for experiments: (*a*) FAPA ion source, (*b*) heating system, (*c*) mass spectrometer inlet

The photograph of the FAPA-MS setup is shown in Fig. 2. The setup consists of a FAPA ion source (Fig. 2a), a heated mini crucible allowing programmable heating in the range of 50–480 °C (Fig. 2b) and the inlet to the mass spectrometer (Fig. 2c). The FAPA ion source was positioned on the axis of the inlet of the mass spectrometer with the tip located ca. 50 mm from the MS inlet. The mini crucible allowing temperature-controlled desorption was placed ca. 10 mm below the ion stream. One milligram of each polymer was placed in a mini crucible and heated from room temperature to ca. 350 °C with a temperature ramp rate of 3 °C s^−1^. The vapors generated in such way were directly introduced into the plasma jet stream, which resulted in their ionization. The mass spectrometer was operating in the positive ion mode during analysis of nicotine or propyphenazone and in the negative ion mode during analysis of methylparaben. The overall average time of analysis was ca. 5 min. For the experiments performed with solutions of the analytes, 10 μL of the appropriate solution were introduced into the mini crucible and heated to ca. 350 °C.

## Results and discussion

### Polymer characterization

**Fig. 3:**
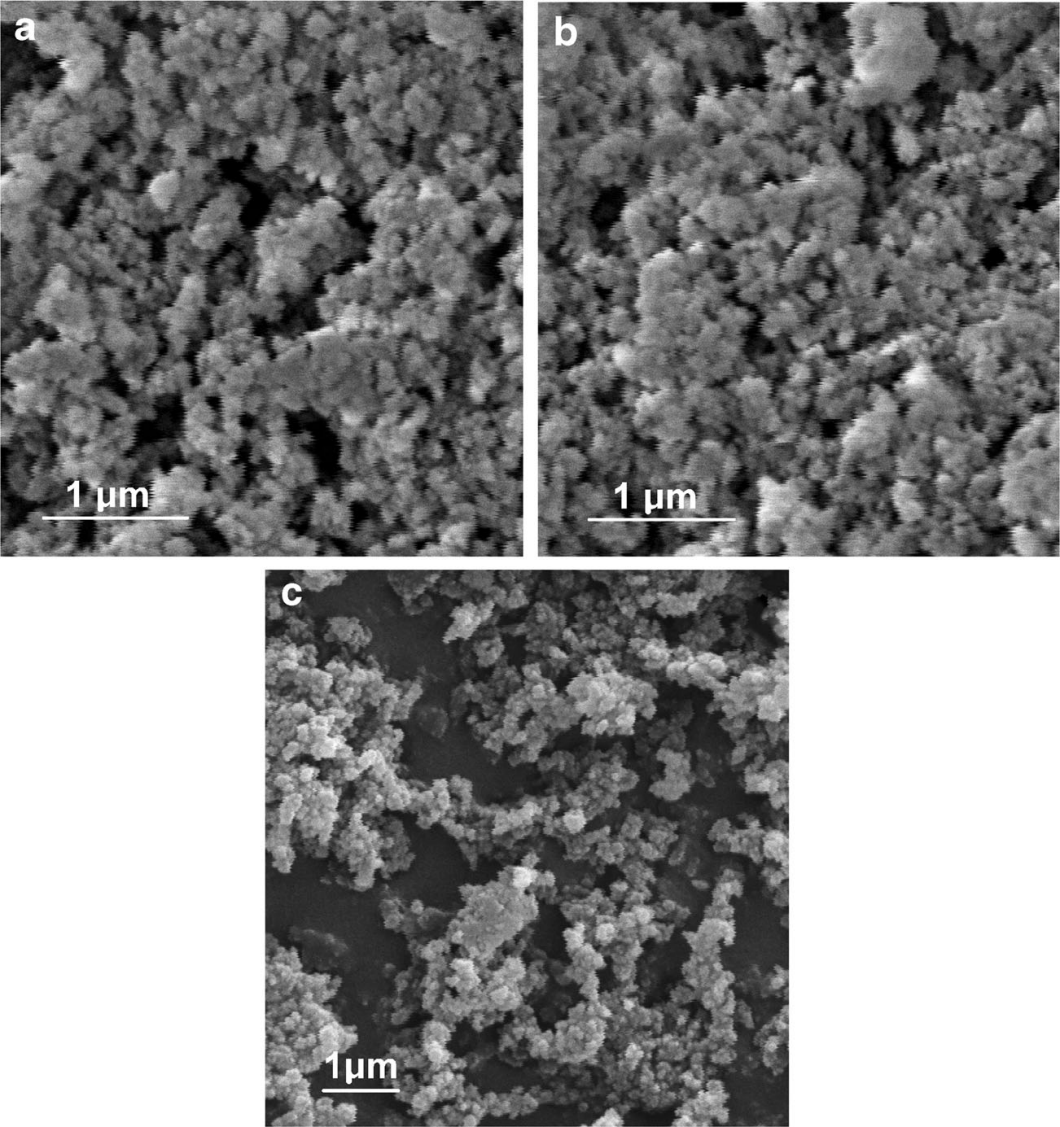
SEM images of **a** leached MIP(nic), **b** non-leached MIP(nic), and **c** NIP

The exemplary SEM images of MIP(nic), MIP(nic) before removal of the template, and NIP are presented in Fig. 3a–c, respectively. All polymer particles have rough surfaces, whereas the distribution of particle sizes is in the range of 0.1–0.4 μm. There are no visible differences in the morphology of MIPs with or without template molecules and NIP. SEM images of MIP(prph) and MIP(mpb) before and after removal of template molecules are presented in Fig. S3 (ESM).

All materials (MIPs with and without template and NIP) display similar characteristic peaks in IR spectra, indicating that the backbone structure of all polymers is similar. The O–H stretching and bending vibrations of carboxyl groups are at 3544 and 1389 cm^−1^, respectively. The symmetric and asymmetric ester C–O stretching vibrations are at 1250 and 1142 cm^−1^, respectively. The stretching vibration of C=O bonds are at 1723 cm^−1^, whereas asymmetric stretching vibrations of CH_2_ groups are at 2955 cm^−1^ [37, 38]. MIPs with template molecules (non-leached) show additional bands characteristic for a particular template: MIP(nic) with nicotine show additional signals at 2779 and 716 cm^−1^; MIP(prph) with propyphenazone show additional signals at 1655, 754, and 698 cm^−1^; MIP(mpb) with methylparaben show additional signals at 1682 and 1316 cm^−1^.

### Adsorption properties of MIPs

**Fig. 4:**
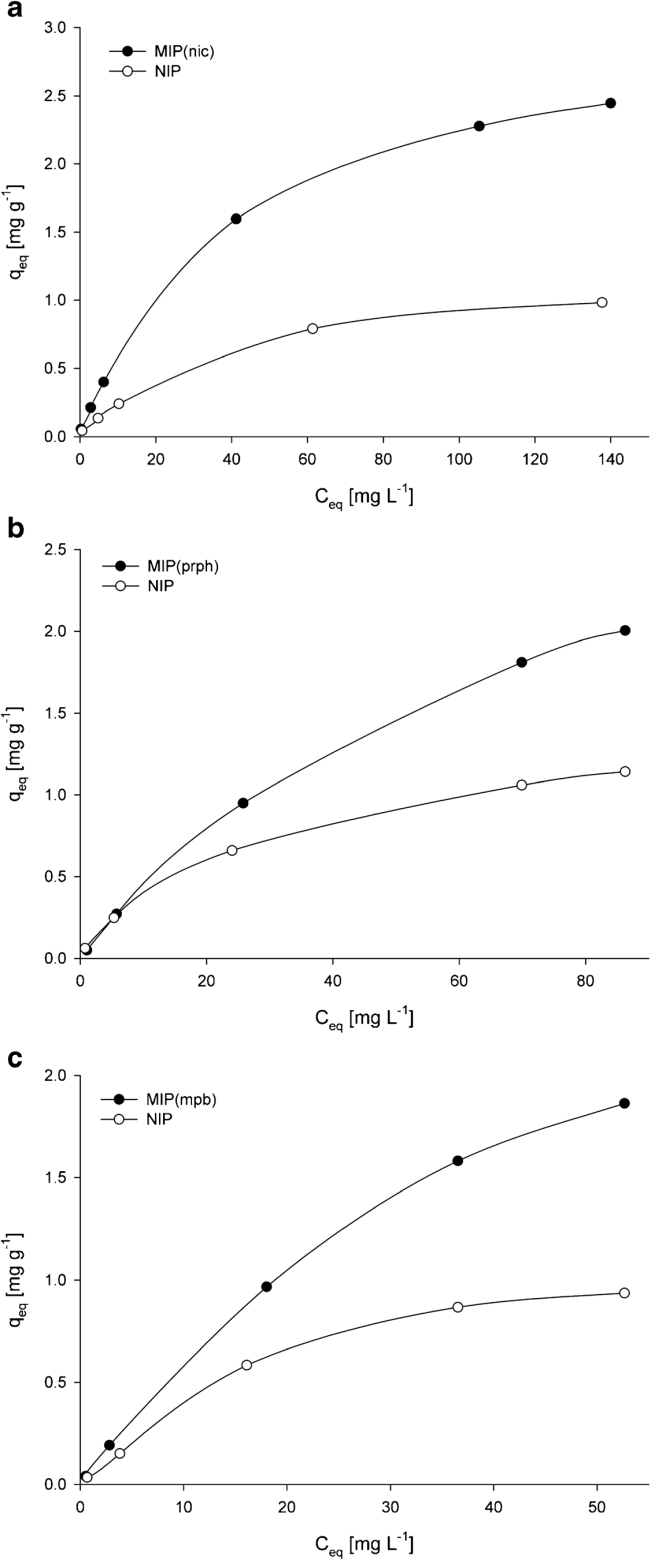
Adsorption isotherms of **a** nicotine, **b** propyphenazone, and **c** methylparaben on the corresponding MIPs and NIP

Adsorption isotherms were used to characterize the adsorption properties of MIPs and NIP at the equilibrium state. Figure 4 presents the relationships between the equilibrium concentration of the analytes and the amount of compounds adsorbed on MIPs or NIP. The adsorption capacities decrease with decreasing initial analyte concentration, which is a result of the lower mass transport coefficient. To describe the adsorption process, Langmuir and Freundlich adsorption isotherm models were considered.

The Langmuir adsorption isotherm was applied in the linearized form:

3 $$ \frac{C_{\mathrm{eq}}}{q_{\mathrm{eq}}}=\frac{C_{\mathrm{eq}}}{q_{\mathrm{m}}}+\frac{1}{K{ q}_{\mathrm{m}}} $$

where *K* (L mg^−1^) is the binding equilibrium constant, *q*_m_ (mg g^−1^) is the maximum amount of the analyte adsorbed, *C*_eq_ (mg L^−1^) is the analyte equilibrium concentration, and *q*_eq_ (mg g^−1^) is the amount of an analyte adsorbed at the concentration *C*_eq_. The calculated values of *q*_m_, *K*, and correlation coefficients (*R* ^2^) are given in Table 1. The *R* ^2^ values obtained for adsorption of the corresponding analytes on MIPs are in the 0.950–0.965 range, which indicates that the experimental data only partially fits the Langmuir adsorption model. On the other hand, the *R* ^2^ values obtained for adsorption on NIP are in the range between 0.970 and 0.987 implying that the data fit the Langmuir adsorption model better than in the case of MIPs. This difference can be explained by the assumptions made in the Langmuir model, particularly that the adsorbed substance forms a monolayer on a completely homogenous surface of the adsorbent. Both MIPs and NIP do not possess completely homogenous surfaces; however, in the case of MIPs, the polymer possesses additional cavities formed during polymerization in the presence of the template. The presence of the cavities is responsible for an increased inhomogeneity of the structure of the adsorbent, reflected by lower *R* ^2^ values. The *q*_m_ values calculated for experiments performed with particular analytes are at least two times higher for MIPs than for NIP. This result clearly indicates that MIPs can absorb more analyte due to distinct imprinting process. The obtained values of *q*_m_ are in accordance with data obtained for other molecularly imprinted polymers [42]. The comparison of binding equilibrium constants (*K* parameters) obtained for MIPs and NIP also suggests an excellent imprinting effect, which is a result of a presence of specific binding sites within the structure of MIPs.

**Table 1:** Parameters of analyte adsorption by MIPs and NIP

Analyte	Adsorbent	Langmuir			Freundlich		
		*q*_m_ (mg g^−1^)	*K* (L mg^−1^)	*R* ^2^	*K*_f_ (mg g^−1^ (L mg^−1^)^1/n^)	1/*n*	*R* ^2^
Nicotine	MIP(nic)	2.86	0.034	0.950	0.127	0.637	0.994
	NIP	1.19	0.033	0.970	0.061	0.583	0.993
Propyphenazone	MIP(prph)	4.23	0.106	0.958	0.051	0.851	0.999
	NIP	1.46	0.043	0.986	0.027	0.620	0.989
Methylparaben	MIP(mpb)	3.88	0.093	0.965	0.074	0.864	0.999
	NIP	1.51	0.031	0.987	0.051	0.759	0.993

The Freundlich adsorption isotherm is mathematically expressed as:

4 $$ {q}_{\mathrm{eq}}={K}_{\mathrm{f}}{C}_{\mathrm{eq}}^{1/ n} $$

5 $$ \log {q}_{\mathrm{eq}}= \log {K}_{\mathrm{f}}+\frac{1}{n} \log {C}_{\mathrm{eq}} $$

where *K*_f_ and *n* represent the Freundlich constants, *C*_eq_ (mg L^−1^) is the analyte equilibrium concentration, and *q*_eq_ (mg g^−1^) is the amount of analyte adsorbed at the concentration *C*_eq_. The calculated values of *K*_f_, 1/*n*, and correlation coefficients (*R* ^2^) are given in Table 1. The *R* ^2^ values obtained for adsorption of corresponding analytes on MIPs are in the 0.994–0.999 range which clearly indicates that the Freundlich isotherm model agrees very well with the experimental data. The Freundlich adsorption model assumes non-ideal adsorption on a heterogeneous surface characterized by uniform energy [43]. Due to these assumptions, this model is more adequate in characterization of analyte adsorption on MIPs. The structure and surface of NIP is more homogenous due to the lack of recognition cavities; therefore, *R* ^2^ values calculated for the Freundlich adsorption model are slightly lower than those of MIPs and are in the 0.989–0.993 range.

**Fig. 5:**
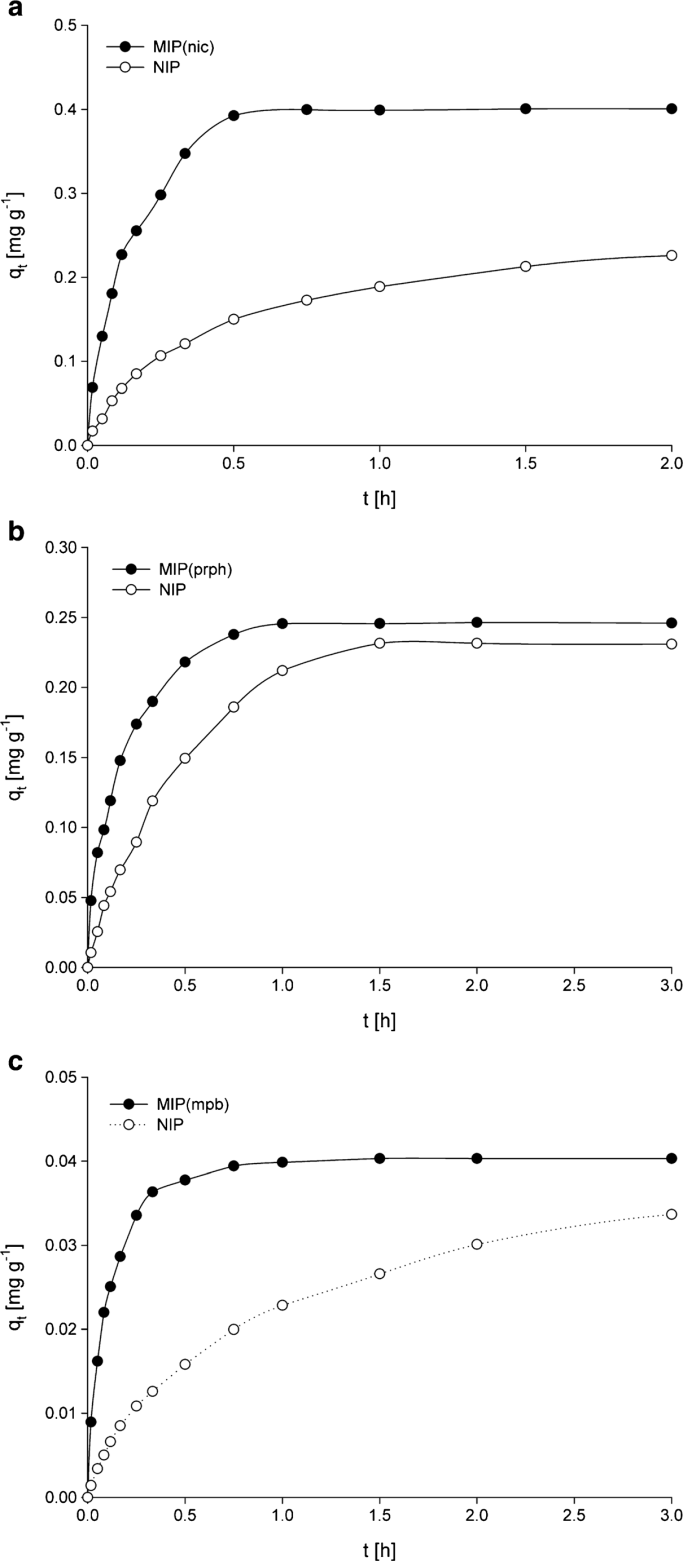
Relationship between time and the adsorption amount for adosprtion of **a** nicotine, **b** propyphenazone, and **c** methylparaben on the corresponding MIPs and NIP

The change in adsorption capacity with interaction time (Fig. 5) was studied to characterize the kinetics of adsorption. Two kinetic models were applied to examine the mechanism of adsorption process. The first model was the pseudo-first-order model given by Langergren and Svenska which is mathematically expressed as:

6 $$ \log \left({q}_{\mathrm{e}}-{q}_{\mathrm{t}}\right)={ \log q}_{\mathrm{e}}-\frac{k_1}{2.303} t $$

where *q*_e_ and *q*_t_ are the amounts of analyte adsorbed (mg g^−1^) at equilibrium and at time *t* (h), respectively, and *k*_1_ (h^−1^) is the rate constant. The second model applied was the pseudo-second-order equation based on the equilibrium adsorption which can be expressed as:

7 $$ \frac{t}{q_{\mathrm{t}}}=\frac{1}{k_2{q}_{\mathrm{e}}^2}+\frac{1}{q_{\mathrm{e}}} t $$

where *k*_2_ (g mg^−1^ h^−1^) is the rate constant of second-order adsorption. The calculated *k*_1_, *k*_2,_ and *R* ^2^ values are presented in Table 2. The *R* ^2^ values obtained for pseudo-first-order model are relatively small providing that this model is inapplicable to describe the adsorption of analytes on MIPs or NIP. On the contrary, *R* ^2^ values obtained for pseudo-second-order model are higher than 0.991 for all MIPs and NIP that clearly indicates that this model is applicable to describe kinetics of the adsorption process. The *k*_2_ rate constants obtained for adsorption of corresponding analytes on MIPs are for all tested substances higher than those obtained for adsorption on NIP. This result shows that adsorption on MIPs is more favorable than adsorption on NIP.

**Table 2:** Kinetic parameters calculated for pseudo-first-order and pseudo-second-order models

Analyte	Adsorbent	Pseudo-first-order kinetic		Pseudo-second-order kinetic	
		*k*_1_ (h^−1^)	*R* ^2^	*k*_2_ (g mg^−1^ h^−1^)	*R* ^2^
Nicotine	MIP(nic)	4.256	0.882	22.93	0.997
	NIP	0.199	0.771	11.38	0.999
Propyphenazone	MIP(prph)	1.708	0.866	16.71	0.991
	NIP	0.177	0.511	14.13	0.999
Methylparaben	MIP(mpb)	3.098	0.936	33.11	0.999
	NIP	1.357	0.955	20.68	0.995

### FAPA-MS experiments

The combination of MIPs and ambient plasma mass spectrometry was achieved by in situ thermal desorption carried in a heated mini crucible. The vapors generated during this process contained an analyte adsorbed by MIPs. Thermal desorption was conducted up to 350 °C, which resulted in decomposition of the polymer. In all experiments, the extracted ion chromatograms (EIC) of the ions corresponding to nicotine ([M + H]^+^ at *m*/*z* 163), propyphenazone ([M + H]^+^ at *m*/*z* 231), and methylparaben ([M − H]^−^ at *m*/*z* 151) have been obtained to exclude ions generated during polymer decomposition. EIC was integrated and the averages of five measurements were used for subsequent calculations. The results of FAPA-MS experiments obtained with the use of MIPs, NIPs, and pure solution of analytes have been compared. Blank experiments were conducted using leached MIPs and NIP and were repeated ten times. The relative standard deviation (RSD) obtained for all data did not exceed 20%. The limit of detection (LOD) was calculated in accordance with the definition: LOD = mean blank value + 3 × standard deviation. The LODs computed for nicotine were 10 nM for MIP(nic), 5 μM for NIP, and 10 μM for nicotine solution. For propyphenazone LODs were 0.5 μM for MIP(prph), 5 μM for NIP, and 10 μM for propyphenazone solution. For methylparaben, LODs were 0.1 μM for MIP(mpb), 5 μM for NIP, and 50 μM for methylparaben solution. In comparison with the measurements performed for analytes, MIPs improve LOD for at least two orders of magnitude and for at least one order of magnitude in comparison with NIP. The highest improvement in LOD was observed for nicotine, particularly in comparison with nicotine solution. MIP(nic) had LOD improved by three orders of magnitude, whereas for NIP, LOD was improved by two orders of magnitude. The graphical presentation of the LOD data is presented in Figs. S4 to S12 (ESM).

**Fig. 6:**
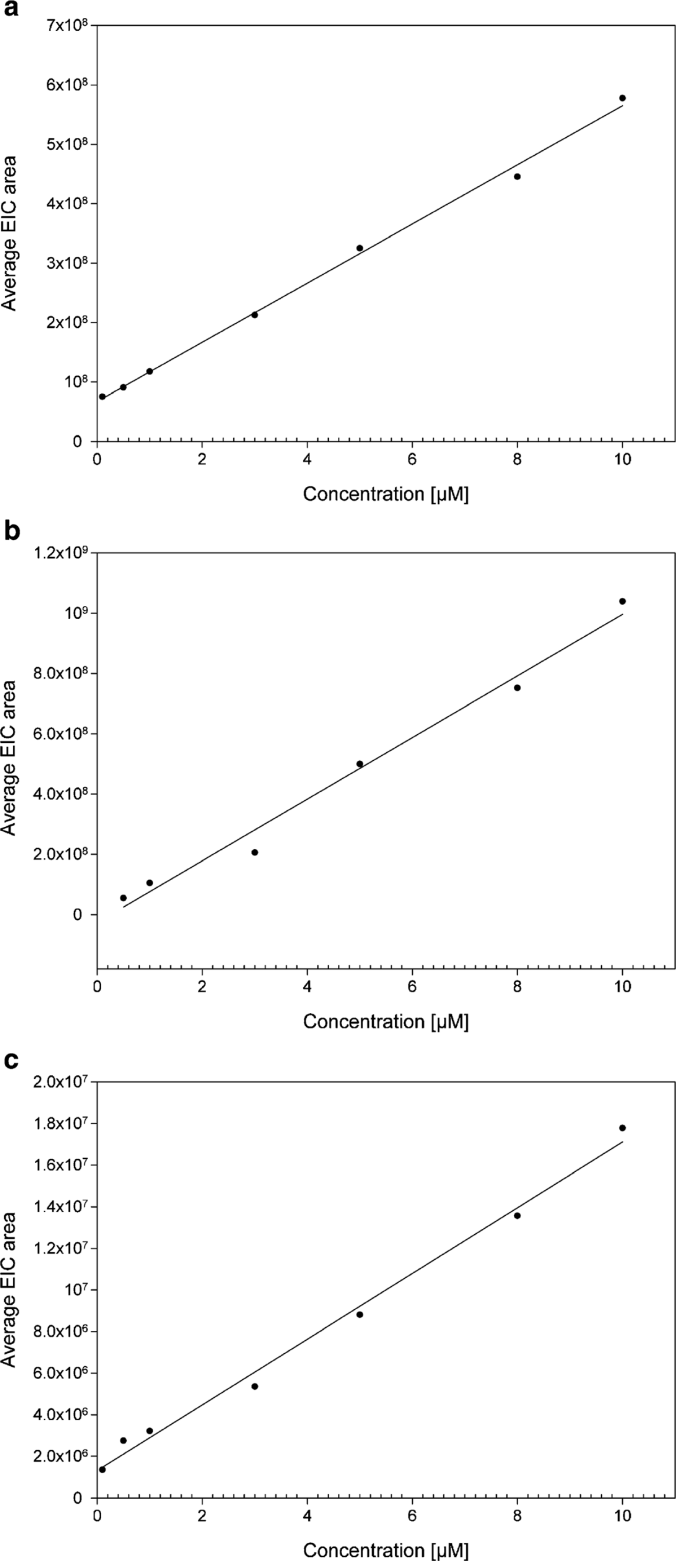
Relationship between average EIC area obtained during FAPA-MS analysis of **a** MIP(nic) and nicotine concentration, **b** MIP(prph) and propyphenazone concentration, and **c** MIP(mpb) and methylparaben concentration

The described procedure has shown linearity in a broad range of concentrations. The experiments performed using MIP(nic) indicated linearity within the range of 0.1 to 10 μM of nicotine (*R* ^2^ = 0.997). MIP(prph) displayed linearity within the range of 0.5 to 10 μM of propyphenazone (*R* ^2^ = 0.986). MIP(mpb) revealed linearity within the range of 0.1 to 10 μM of methylparaben (*R* ^2^ = 0.992). The graphs presenting the relationship between the analyte concentration and an average area obtained after EIC integration for MIP(nic), MIP(prph), and MIP(mpb) are shown in Fig. 6. The lack of linearity at higher concentrations is presumably caused by the saturation either of the ion trap (excess of ions) or MIPs (saturated capacity of the adsorbent).

To evaluate the selectivity of the MIPs, MIP(prph) and MIP(mpb) were immersed in a mixture of their analytes and compounds structurally related to them. MIP(prph) (10 mg) was added to a solution of propyphenazone (1 μM) and phenazone (1 μM). During FAPA-MS analysis, EIC of the ions corresponding to these compounds ([M + H]^+^) have been recorded. Similarly, MIP(mpb) (10 mg) was added to a solution of methylparaben (1 μM), ethylparaben (1 μM), and butylparaben (1 μM). During FAPA-MS analysis, EIC of the ions corresponding to these compounds ([M − H]^−^) have been recorded. Identical set of experiments was performed with the use of NIP. All EICs have been integrated, and the quotients obtained by dividing the areas calculated for the analytes by areas of compounds structurally related to them are given in Table 3. In both experiments involving the use of MIPs, a clear increase in selectivity towards the analyte molecules can be observed. This indicates that MIPs show an increased affinity towards target molecules and selectively interact with them.

**Table 3:** Results of selectivity experiments of MIPs and NIP

Compound	NIP	MIP(prph)	MIP(mpb)
Propyphenazone/phenazone	0.406	3.938	–
Methylparaben/ethylparaben	0.223	–	0.471
Methylparaben/butylparaben	0.148	–	0.309

Data presented are quotients obtained by dividing EIC areas (after integration) measured for the compounds given

### Analysis of real samples

To validate the performance of the proposed method for determination of nicotine, propyphenazone, and methylparaben in the real-life samples, urine and plasma samples were analyzed and the results obtained are collected in Table 4. To evaluate the applicability of the analytical results, urine and plasma samples were spiked with the analytes at various concentrations. Quantification was done using appropriate MIPs in combination with FAPA-MS analysis. The resulting recovery values (94.6–98.4%) show good performance of the proposed combination of MIPs and FAPA-MS and confirm that this method can be applied for the analysis of various analytes in the real-life samples.

**Table 4:** Analytical results obtained for real-life samples

Sample	Analyte	Added [μM]	Found [μM]	Recovery [%]
Urine	Nicotine	5.00	4.73	94.6
Urine	Nicotine	0.50	0.48	96.0
Plasma	Propyphenazone	5.00	4.92	98.4
Plasma	Propyphenazone	1.00	0.95	95.0
Urine	Methylparaben	5.00	4.81	96.2
Urine	Methylparaben	1.00	0.97	97.0

### Comparison with other methods

To show the performance of the proposed combination of MIPs and FAPA-MS, the technique has been compared to other previously reported methods for determination of nicotine, propyphenazone, and methylparaben in terms of LOD and linearity (Table 5). The developed method has lower LOD and improved linearity than other reported techniques used for detection and quantification of nicotine or propyphenazone. In comparison with procedures used for detection and quantification of methylparaben, the developed setup has lower LOD than pulse voltammetry, however, higher than an ultrahigh performance liquid chromatography-tandem mass spectrometry coupled with dispersive liquid-liquid microextraction [44]. The latter method is time-consuming as compared to the 5-min analysis time.

**Table 5:** Comparison with other analytical methods

Analyte	Technique	LOD	Linearity	Reference
Nicotine	Voltammetry	0.866 μM	1–200 μM	[45]
Nicotine	MIP + heat-transfer method	0.1 μM	0.1–2.5 μM	[46]
Nicotine	MIP + FAPA-MS	10 nM	0.1–10 μM	This work
Propyphenazone	Sequential injection chromatography	3 μM	0.01–1.3 mM	[47]
Propyphenazone	Electrokinetic capillary chromatography	3.47 μM	0.013–0.868 mM	[48]
Propyphenazone	MIP + FAPA-MS	0.5 μM	0.5–10 μM	This work
Methylparaben	Pulse voltammetry	8 μM	0.01–5 mM	[49]
Methylparaben	UHPLC − MS/MS	1.31 nM	0.004–0.33 μM	[44]
Methylparaben	MIP + FAPA-MS	0.1 μM	0.1–10 μM	This work

## Conclusions

In summary, we have shown that molecularly imprinted polymers can be effectively used as selective adsorbents prior to the analysis by ambient plasma mass spectrometry. Synthesis of the MIPs is simple, involves low-cost reagents, and results in the structures that possess high adsorption properties combined with good selectivity. A simple setup with heating system with programmable temperature allows for thermal desorption of the analytes from MIPs to be directly introduced into FAPA ion stream. The developed method offers rapid analysis, improved LOD over other analytical methods, and linearity for broad range of concentrations. The estimated limits of detection clearly indicate that the presented analytical procedure allows for detection of organic compounds at very low concentrations, making such technique also suitable for diagnostic purposes. The developed method is a destructive analytical technique, as the MIPs undergo thermal decomposition; therefore, their reuse in not possible. Taking into account the low cost of their synthesis, this issue can be considered as minor. The combination of MIPs and FAPA-MS has proven to be effective in analysis of real-life samples such as the urine and plasma. Further improvement of these limits of detection by using MS/MS analysis or single ion monitoring is currently in progress in our laboratory.

## Electronic supplementary material

Below is the link to the electronic supplementary material.

ESM 1 (PDF 2707 kb)
