# Supplementary material for: Molecularly imprinted polymers as selective adsorbents for ambient plasma mass spectrometry
Source: Anal Bioanal Chem. 2017 Mar 20;409(13):3393–405. doi: 10.1007/s00216-017-0281-2 (PMC5395590; doi:10.1007/s00216-017-0281-2)
Supplement: Supplementary file 1 — (PDF 2707 kb) [file 216_2017_281_MOESM1_ESM.pdf]

Analytical and Bioanalytical Chemistry

Electronic Supplementary Material

Molecularly imprinted polymers as selective adsorbents for ambient plasma mass spectrometry

Michał Cegłowski, Marek Smoluch, Edward Reszke, Jerzy Silberring, Grzegorz Schroeder

Table of contents

Fig. S1 A scheme of MIP(prph) synthesis S-3

Fig. S2 A scheme of MIP(mpb) synthesis S-4

Fig. S3 SEM images of (a) leached MIP(prph); (b) non-leached MIP(prph),; (c) leached MIP(mpb), and (d) non-leached MIP(mpb) S-5

Fig. S4 Results from LOD experiments for MIP(nic); the red dashed line represents LOD value (mean blank value + 3 × standard deviation) S-6

Fig. S5 Results from LOD experiments for NIP + nicotine; the red dashed line represents LOD value (mean blank value + 3 × standard deviation) S-7

Fig. S6 Results from LOD experiments for nicotine solution; the red dashed line represents LOD value (mean blank value + 3 × standard deviation) S-8

Fig. S7 Results from LOD experiments for MIP(prph); the red dashed line represents LOD value (mean blank value + 3 × standard deviation) S-9

Fig. S8 Results from LOD experiments for NIP + propyphenazone; the red dashed line represents LOD value (mean blank value + 3 × standard deviation) S-10

Fig. S9 Results from LOD experiments for propyphenazone solution; the red dashed line represents LOD value (mean blank value + 3 × standard deviation) S-11

Fig. S10 Results from LOD experiments for MIP(mpb); the red dashed line represents LOD value (mean blank value + 3 × standard deviation) S-12

Fig. S11 Results from LOD experiments for NIP + methylparaben; the red dashed line represents LOD value (mean blank value + 3 × standard deviation) S-13

Fig. S12 Results from LOD experiments for methylparaben solution; the red dashed line represents LOD value (mean blank value + 3 × standard deviation) S-14

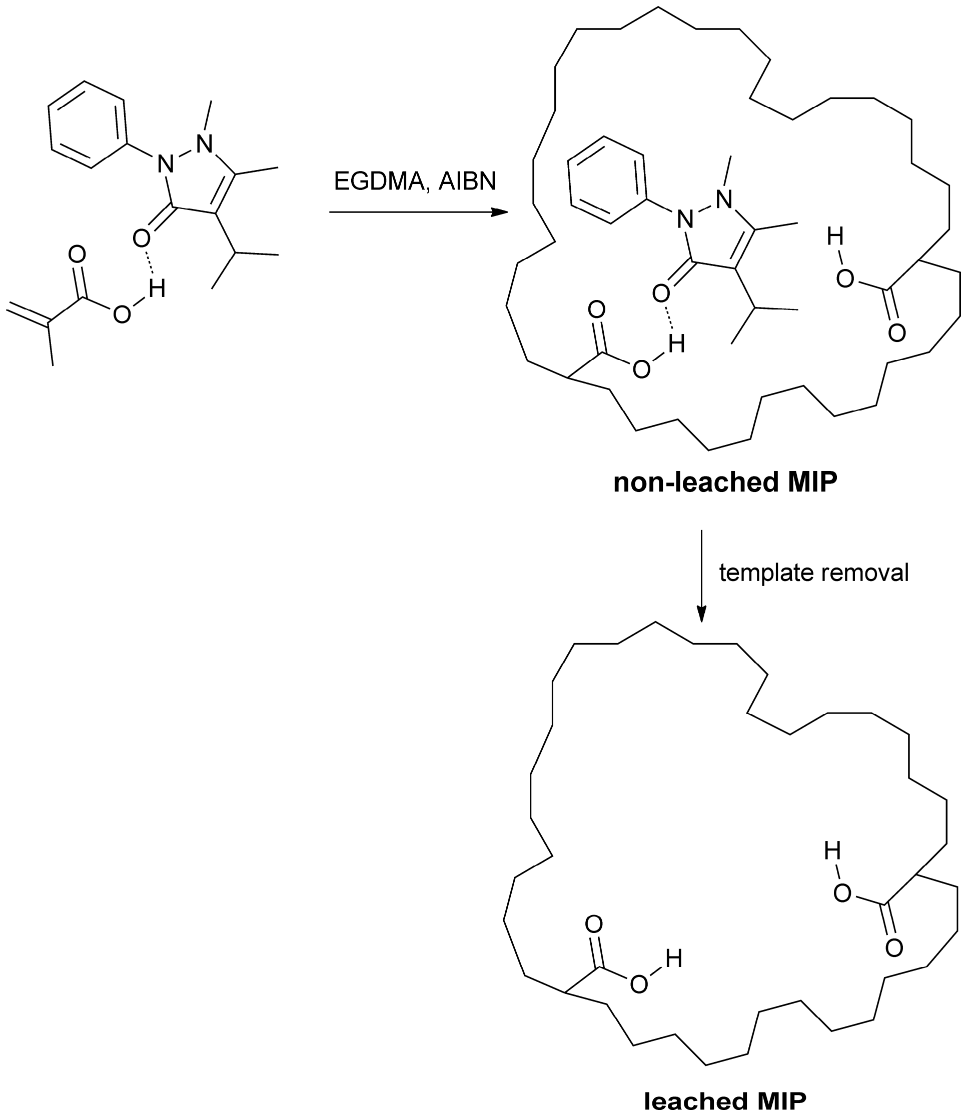

Fig. S1 A scheme of MIP(prph) synthesis

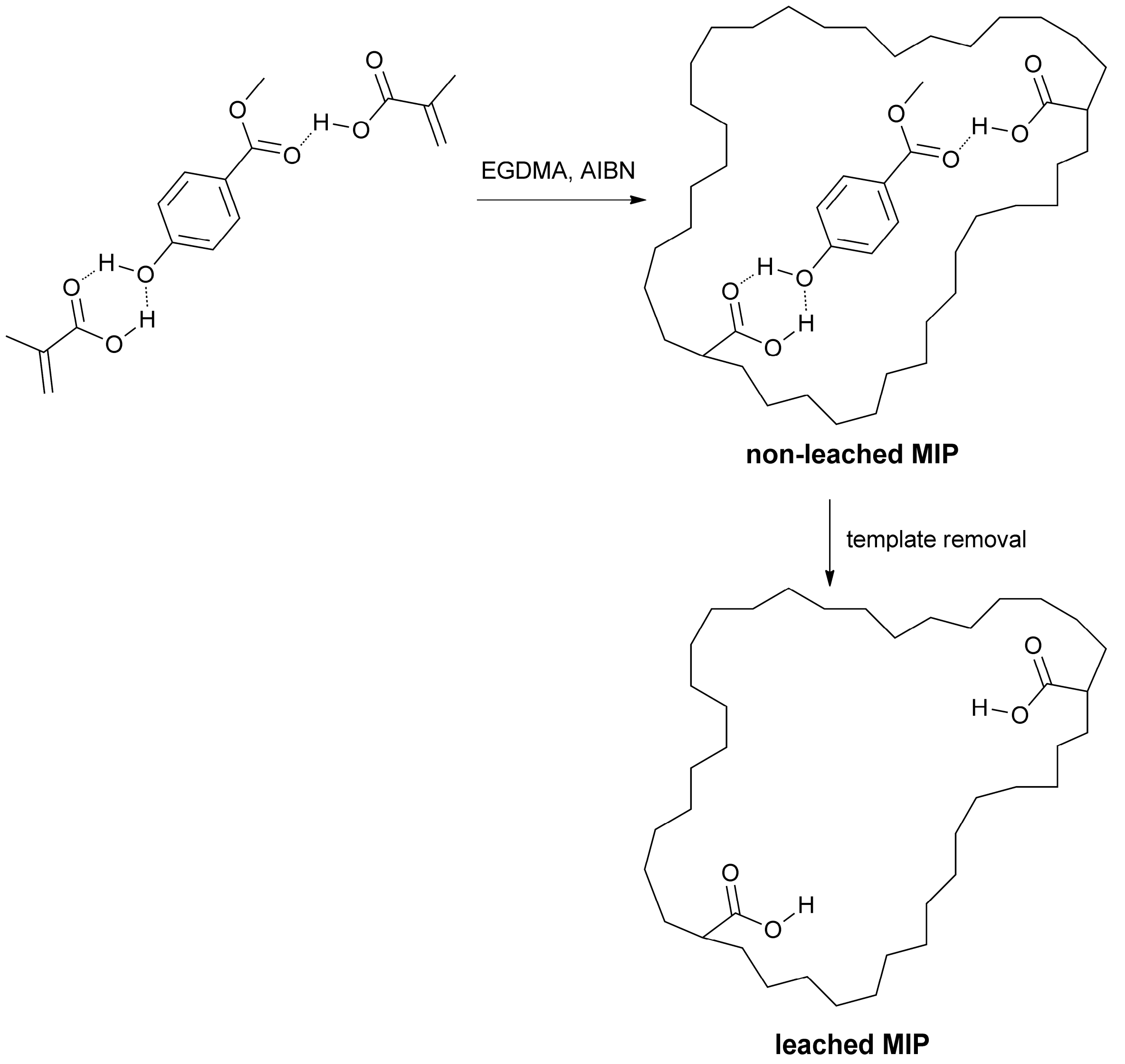

Fig. S2 A scheme of MIP(mpb) synthesis

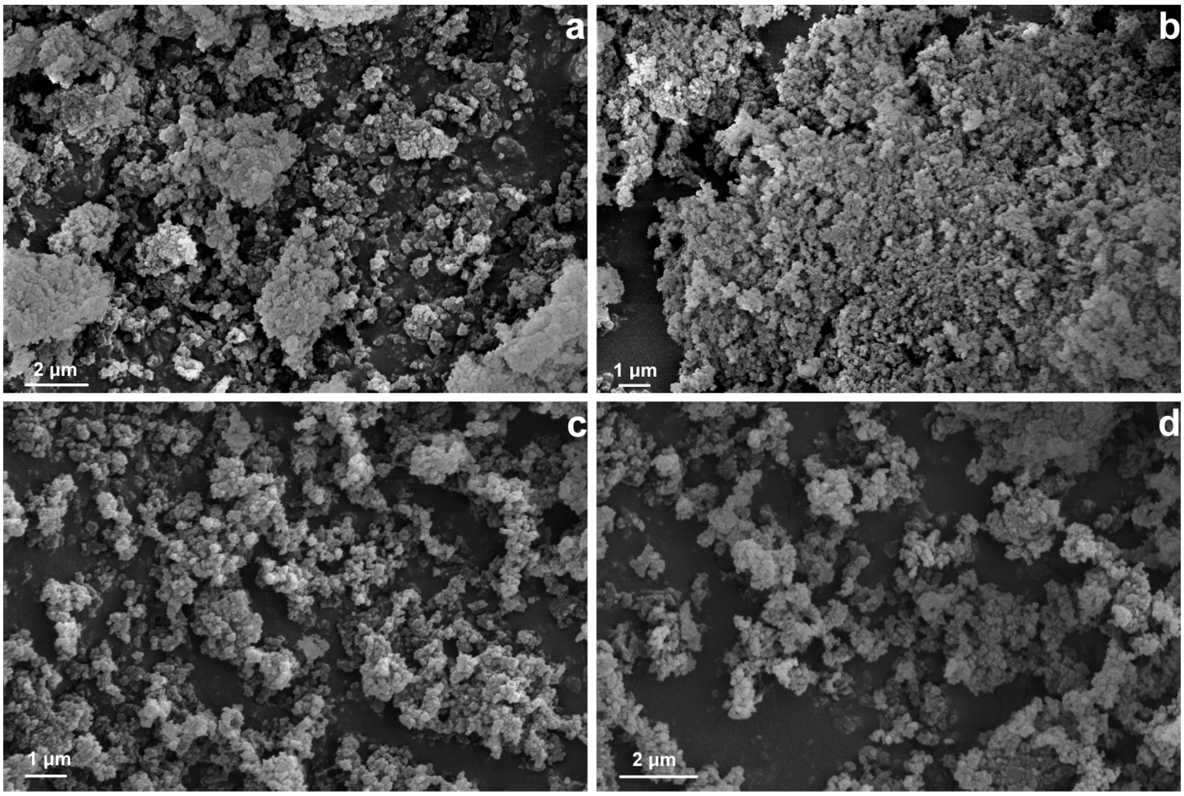

Fig. S3 SEM images of (a) leached MIP(prph); (b) non-leached MIP(prph); (c) leached MIP(mpb), and (d) non-leached MIP(mpb)

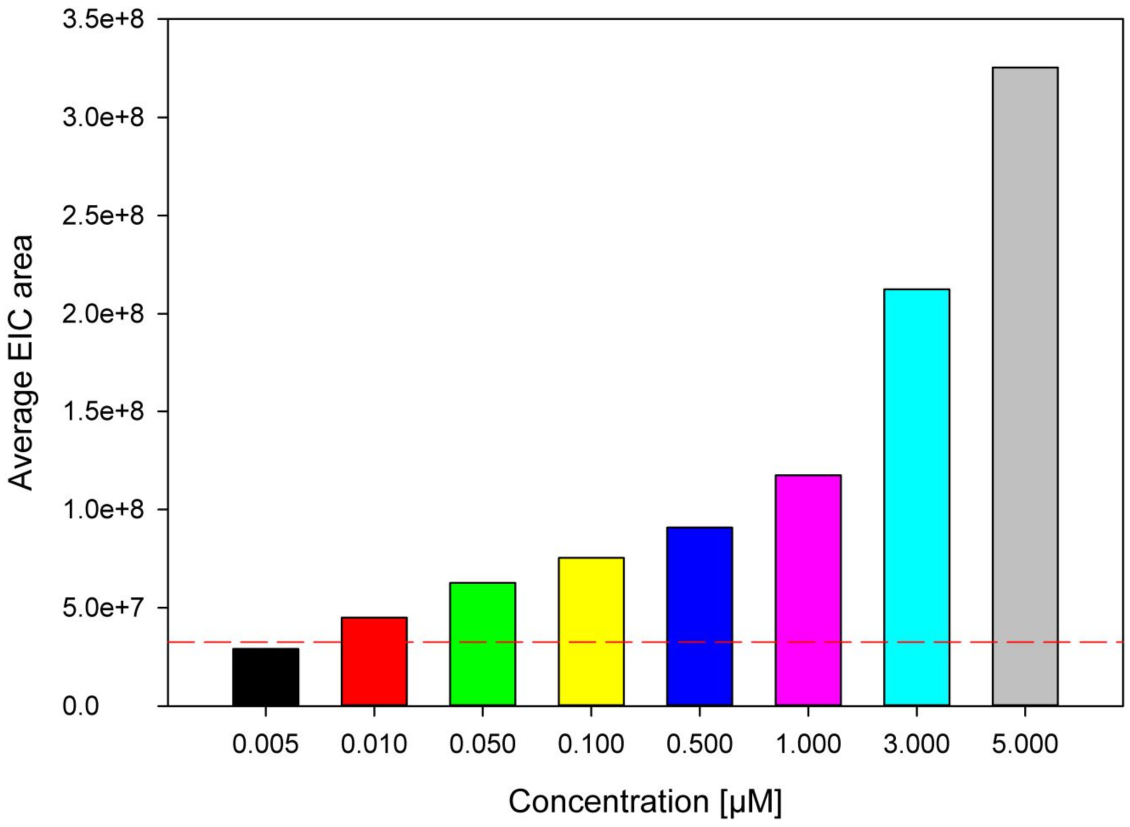

Fig. S4 Results from LOD experiments for MIP(nic); the red dashed line represents LOD value (mean blank value + 3 × standard deviation)

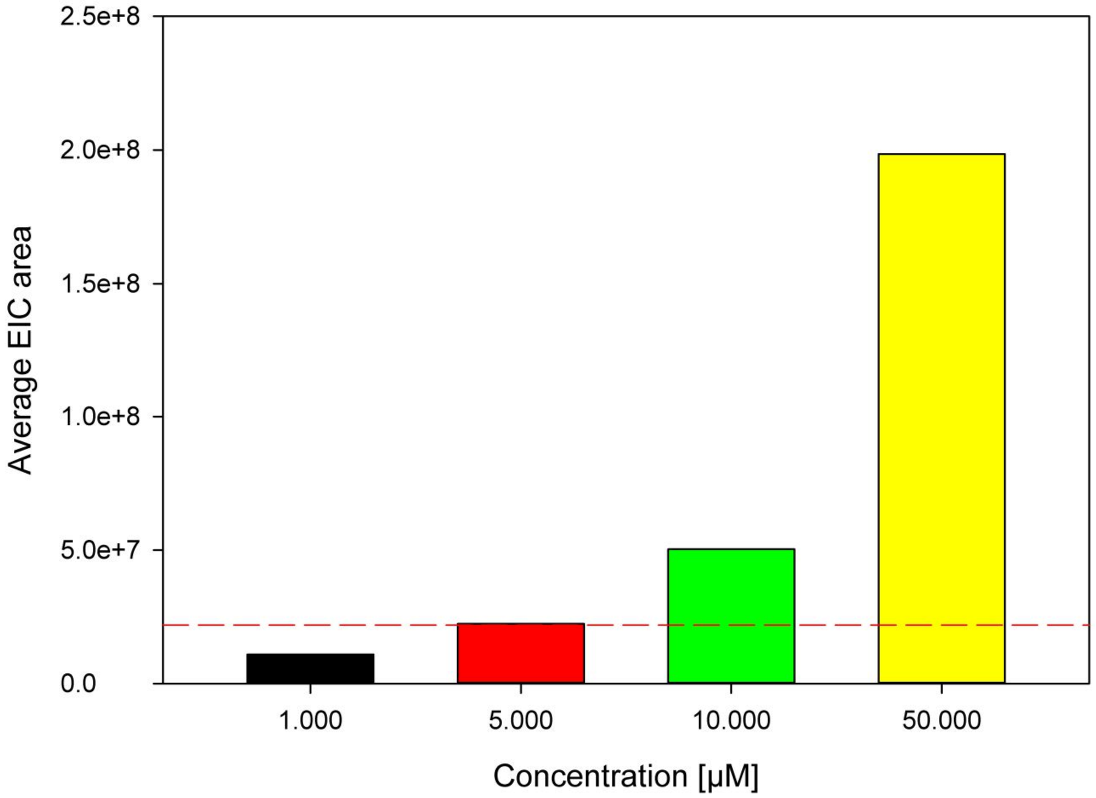

Fig. S5 Results from LOD experiments for NIP + nicotine; the red dashed line represents LOD value (mean blank value + 3 × standard deviation)

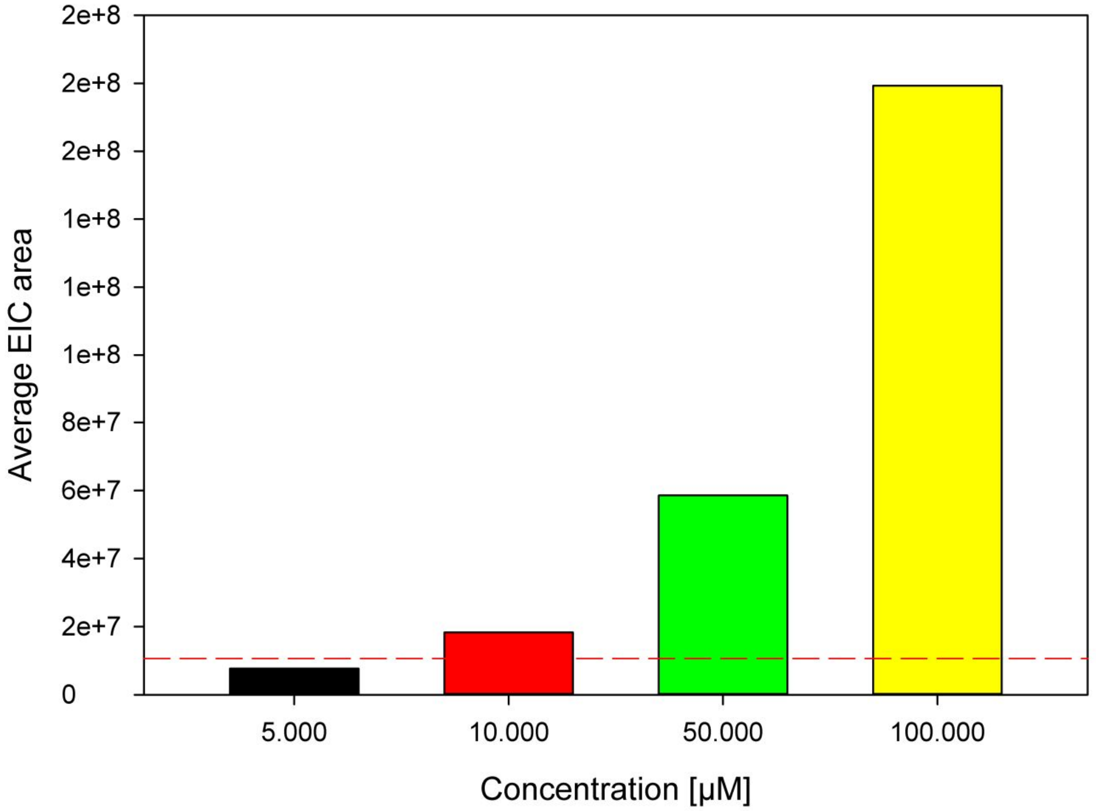

Fig. S6 Results from LOD experiments for nicotine solution; the red dashed line represents LOD value (mean blank value + 3 × standard deviation)

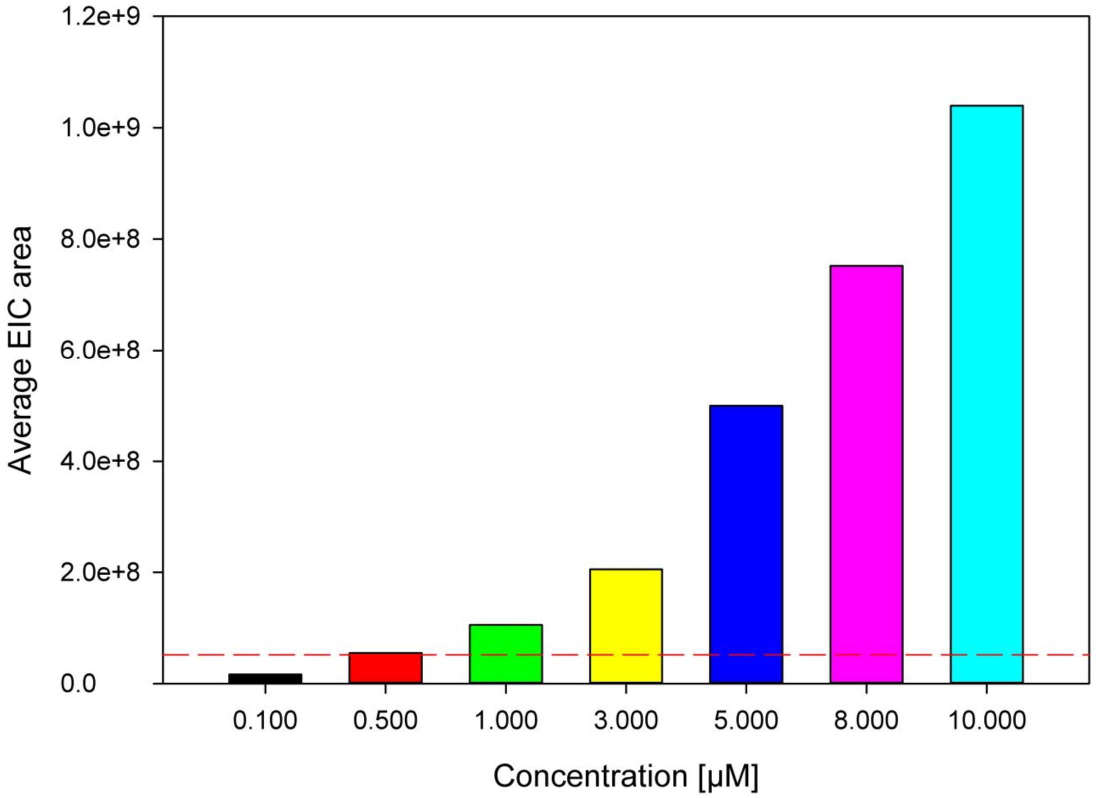

Fig. S7 Results from LOD experiments for MIP(prph); the red dashed line represents LOD value (mean blank value + 3 × standard deviation)

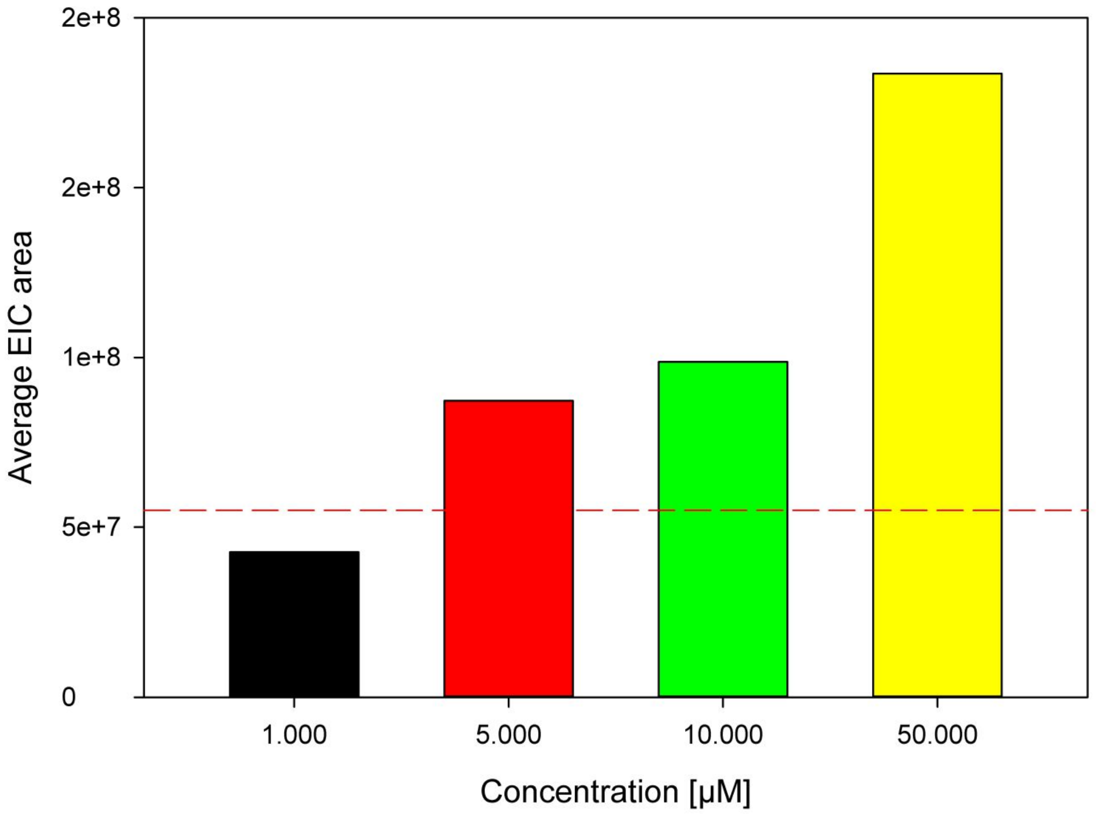

Fig. S8 Results from LOD experiments for NIP + propyphenazone; the red dashed line represents LOD value (mean blank value + 3 × standard deviation)

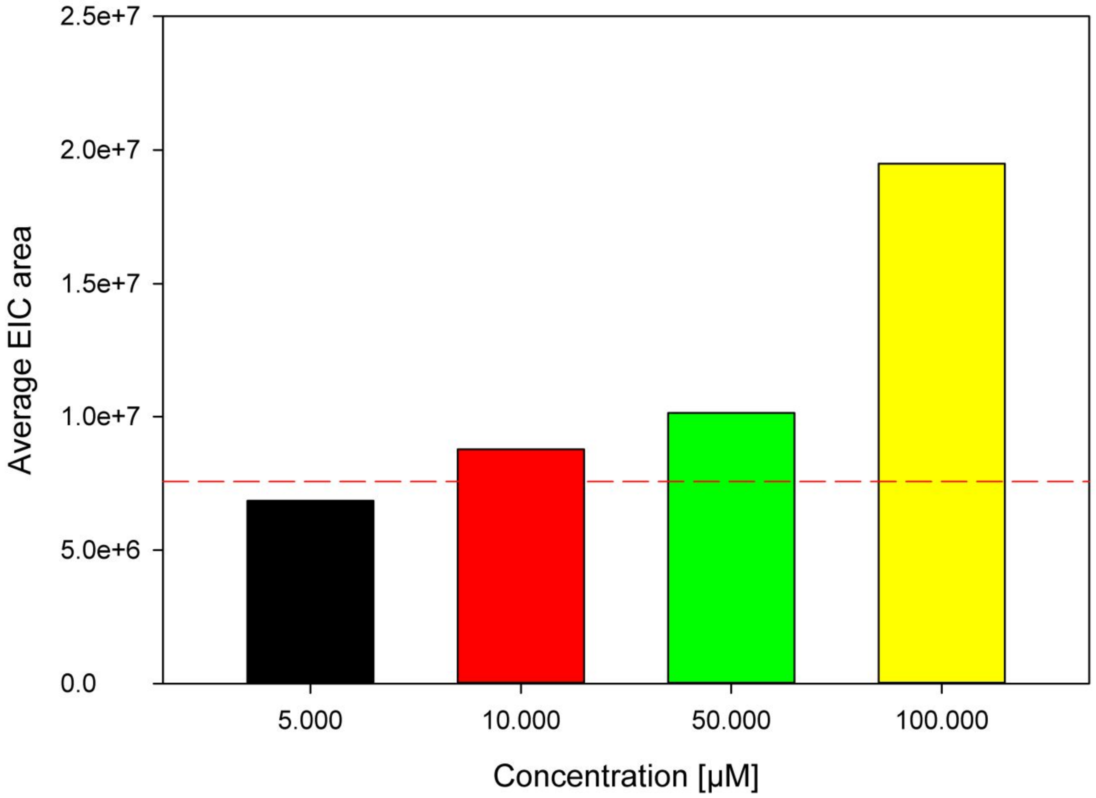

Fig. S9 Results from LOD experiments for propyphenazone solution; the red dashed line represents LOD value (mean blank value + 3 × standard deviation)

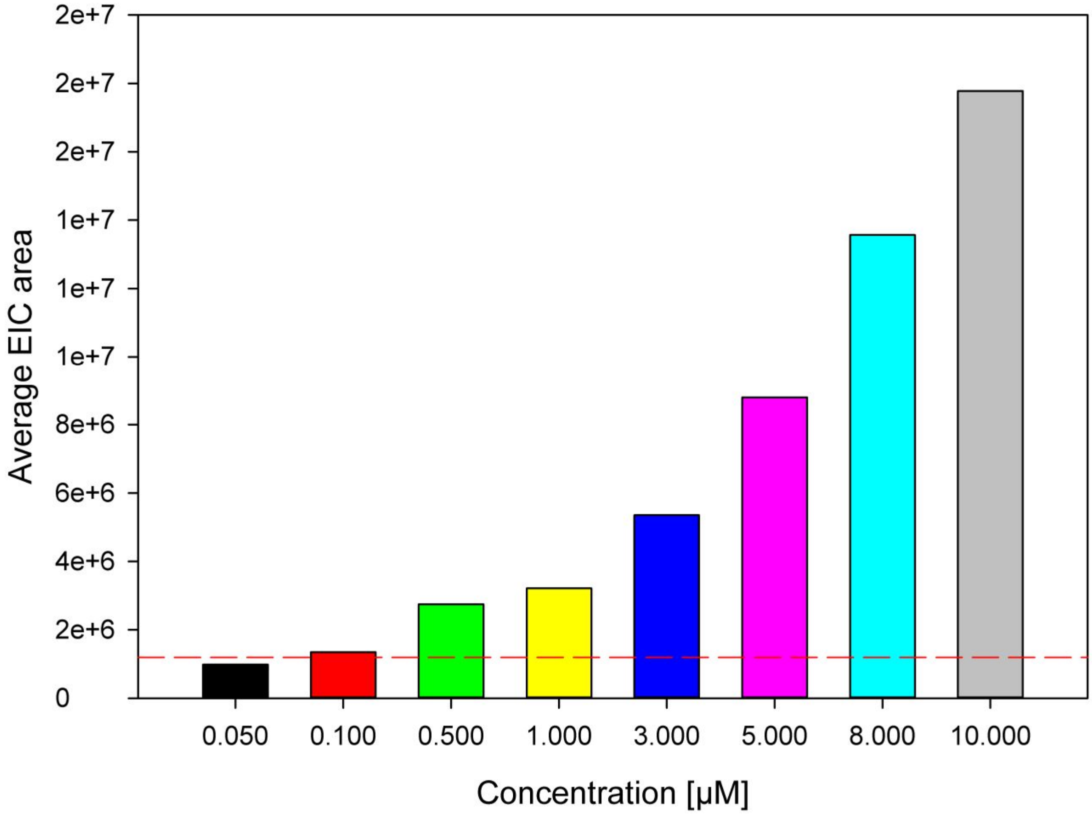

Fig. S10 Results from LOD experiments for MIP(mpb); the red dashed line represents LOD value (mean blank value + 3 × standard deviation)

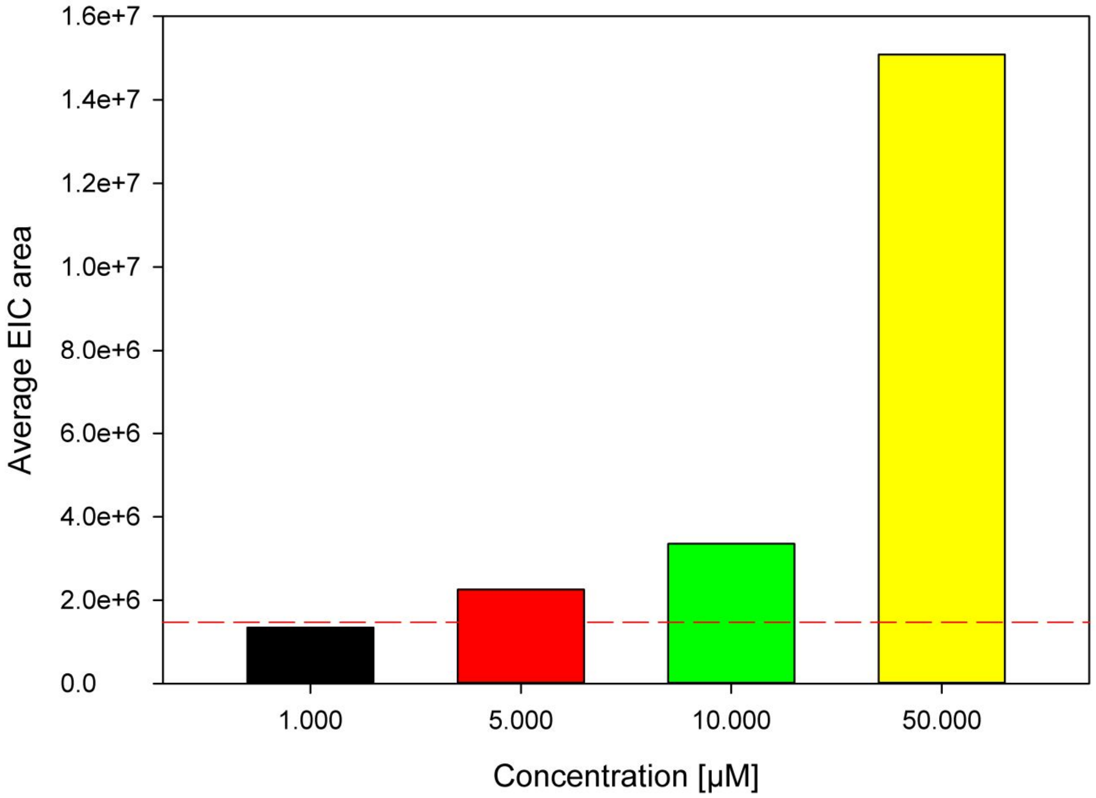

Fig. S11 Results from LOD experiments for NIP + methylparaben; the red dashed line represents LOD value (mean blank value + 3 × standard deviation)

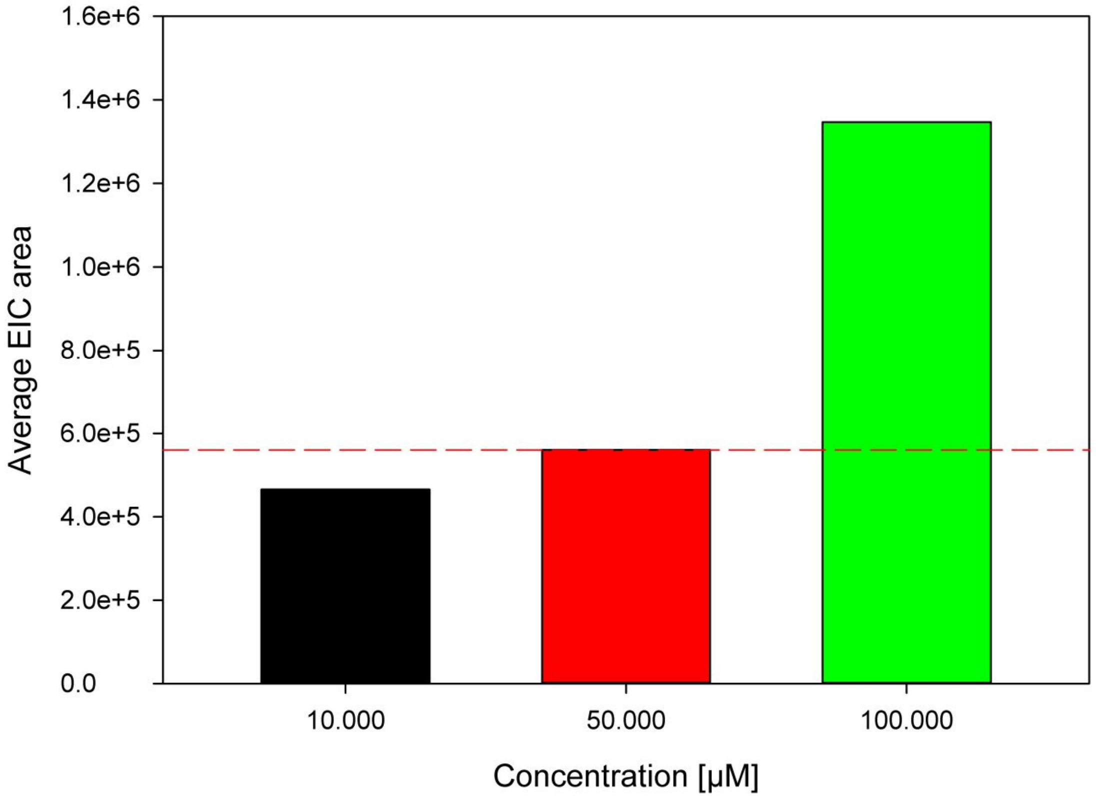

Fig. S12 Results from LOD experiments for methylparaben solution; the red dashed line represents LOD value (mean blank value + 3 × standard deviation)
